# Do State Agency on Aging Strategic Plans Include Terms Related to Malnutrition?

**DOI:** 10.1093/geroni/igab046.3265

**Published:** 2021-12-17

**Authors:** Mary Beth Arensberg, Jaime Gahche, Johanna Dwyer

**Affiliations:** 1 Abbott, Columbus, Ohio, United States; 2 National Institutes of Health, Bethesda, Maryland, United States; 3 National Institutes of Health, National Institutes of Health, Maryland, United States

## Abstract

Demand for federal nutrition assistance programs is increasing as the older population grows and further accelerated with the COVID-19 pandemic. Older adult nutrition programs are based on federal nutrition guidelines that have traditionally focused on healthy populations, yet many older adults have multiple chronic conditions/advanced age. Some guidelines are changing; the 2020 Dietary Guidelines for Americans recognize older adults’ risk for malnutrition and also need for adequate protein to prevent lean muscle loss with age. The 2020 Older Americans Act (OAA) reauthorization included reduction of malnutrition in OAA’s official purpose and added program participant screening for malnutrition. The OAA requires State Agencies on Aging submit multiyear strategic plans to receive program funding, but it is unknown how the plans address risks for malnutrition, including overweight, underweight, and muscle loss (sarcopenia/frailty). We searched 51 State Agency on Aging strategic plans posted at advancingstates.org to determine their frequency of mentioning nutrition, malnutrition/underweight/undernutrition, obesity/overweight, frail/frailty, sarcopenia, and dietary supplements/oral nutrition supplements (DS/ONS)/meal replacements. Every state plan included nutrition but less than a third included malnutrition. There was wide variability in how nutrition and malnutrition were incorporated into state goals and strategies. Very few plans included obesity, frailty, and DS/ONS terms; none included sarcopenia. Although there has been some movement, there is need for many State Agencies on Aging plans to address all aspects of malnutrition including overweight, underweight/other factors related to muscle loss (sarcopenia/frailty) that adversely impact healthy aging. Wide disparities in plan structure/use of terms create opportunities for more common approaches/definitions.

